# Orexin-1 receptor blockade dysregulates REM sleep in the presence of orexin-2 receptor antagonism

**DOI:** 10.3389/fnins.2014.00028

**Published:** 2014-02-14

**Authors:** Christine Dugovic, Jonathan E. Shelton, Sujin Yun, Pascal Bonaventure, Brock T. Shireman, Timothy W. Lovenberg

**Affiliations:** Neuroscience, Janssen Research & Development, L.L.C.San Diego, CA, USA

**Keywords:** orexin-1, orexin-2, receptor antagonist, REM sleep, rat

## Abstract

In accordance with the prominent role of orexins in the maintenance of wakefulness via activation of orexin-1 (OX1R) and orexin-2 (OX2R) receptors, various dual OX1/2R antagonists have been shown to promote sleep in animals and humans. While selective blockade of OX2R seems to be sufficient to initiate and prolong sleep, the beneficial effect of additional inhibition of OX1R remains controversial. The relative contribution of OX1R and OX2R to the sleep effects induced by a dual OX1/2R antagonist was further investigated in the rat, and specifically on rapid eye movement (REM) sleep since a deficiency of the orexin system is associated with narcolepsy/cataplexy based on clinical and pre-clinical data. As expected, the dual OX1/2R antagonist SB-649868 was effective in promoting non-REM (NREM) and REM sleep following oral dosing (10 and 30 mg/kg) at the onset of the dark phase. However, a disruption of REM sleep was evidenced by a more pronounced reduction in the onset of REM as compared to NREM sleep, a marked enhancement of the REM/total sleep ratio, and the occurrence of a few episodes of direct wake to REM sleep transitions (REM intrusion). When administered subcutaneously, the OX2R antagonist JNJ-10397049 (10 mg/kg) increased NREM duration whereas the OX1R antagonist GSK-1059865 (10 mg/kg) did not alter sleep. REM sleep was not affected either by OX2R or OX1R blockade alone, but administration of the OX1R antagonist in combination with the OX2R antagonist induced a significant reduction in REM sleep latency and an increase in REM sleep duration at the expense of the time spent in NREM sleep. These results indicate that additional blockade of OX1R to OX2R antagonism elicits a dysregulation of REM sleep by shifting the balance in favor of REM sleep at the expense of NREM sleep that may increase the risk of adverse events. Translation of this hypothesis remains to be tested in the clinic.

## Introduction

The orexin neuropeptides produced by lateral hypothalamic neurons play a critical role in the maintenance of wakefulness by activating two distinct receptors, the orexin-1 (OX1R) and the orexin-2 (OX2R) receptor that are widely distributed throughout the brain (De Lecea et al., 1998; Peyron et al., 1998; Sakurai et al., 1998). The orexin system is believed to stabilize the wake-sleep flip-flop switch in wake-active structures consisting of histaminergic, monoaminergic, and cholinergic neurons (Saper et al., 2001), and also to regulate the onset of rapid eye movement (REM) sleep and associated muscular atonia in the brainstem (Lu et al., 2006). In accordance with the prominent function of orexins in sustaining wakefulness, pharmacological blockade of both OX1R and OX2R (OX1/2R) has been shown to promote sleep in various species, and the dual OX1/2R antagonists almorexant, SB-649868 and suvorexant have been clinically validated for the treatment of insomnia (Winrow and Renger, 2014). Further investigations conducted in rodent models on the specific role of OX1R and OX2R in sleep modulation indicate that while selective blockade of OX2R seems to be sufficient to initiate and prolong sleep (Dugovic et al., 2009; Mang et al., 2012), the beneficial effect of additional inhibition of OX1R remains controversial (Morairty et al., 2012).

OX1R and OX2R are differentially distributed in structures regulating sleep and wake, with OX1R exclusively expressed in the locus coeruleus, OX2R selectively expressed in the tuberomammillary nucleus, and both receptors co-expressed in the dorsal raphe (Sakurai et al., 1998; Marcus et al., 2001), suggesting a distinct function between the two orexin receptors. It has been proposed that OX2R signaling is essential for the promotion of wakefulness and the transition to non-REM (NREM) sleep and that both OX1R and OX2R contribute to REM sleep suppression (Willie et al., 2003; Mieda et al., 2011; Mochizuki et al., 2011). Loss or disruption of orexin signaling in human and animal narcolepsy is associated with reduced activity of the wake-promoting system, frequent transitions into NREM sleep, and abnormal intrusions of REM sleep into wake accompanied by loss of muscular tone and cataplexy (Nishino, 2007). During their dark/active phase, mice lacking the orexin peptide as well as mice lacking both OX1R and OX2R spend more time in REM sleep, whereas NREM sleep duration is unaffected as compared to their corresponding wild type (Willie et al., 2003; Sakurai, 2007; Mang et al., 2012). Previous work in our lab has indicated that simultaneous blockade of OX1R attenuates the NREM sleep-promoting effects evoked by a selective OX2R antagonist but not the REM sleep promotion when assessed for a 2-h period during the light/rest phase in rats (Dugovic et al., 2009).

In the present study, we further explored the respective contributions of OX1R and OX2R on the sleep-promoting effects elicited by pharmacological blockade of both receptors, and specifically on REM sleep since a deficiency of the orexin system is associated with narcolepsy/cataplexy based on clinical and pre-clinical data. In order to detect possible events reminiscent of narcoleptic-like symptoms such as REM intrusion into wakefulness, the investigation was conducted during the active phase of the animals. The effects of the dual OX1/2R antagonist SB-649868 (Di Fabio et al., 2011) were compared to those obtained by co-administration of the selective OX1R antagonist GSK-1059865 (Gozzi et al., 2011) and the selective OX2R antagonist JNJ-10397049 (McAtee et al., 2004) during the dark phase in rats.

## Materials and methods

### Animals

Studies were performed in male Sprague–Dawley rats (Harlan Laboratories, weighing 350–450 g). Animals were approximately 4 months of age at the start of the study and were housed individually in cages under controlled conditions with lights on at 6 AM (12:12 light/dark schedule) while temperature was maintained at 22 ± 2°C. During the course of the study, animals had *ad libitum* access to food and water. All procedures detailed in this investigation were implemented in accordance with policies established by the Guide for the Care and Use of Laboratory Animals as adopted by the United States National Institutes of Health.

### Drugs and experimental design

SB-649868 is a dual OX1/2R antagonist with similar potency at both receptor subtypes (pKi OX1R = 9.5, pKi OX2R = 9.4) and the doses tested were selected in accordance with the pharmacokinetic profile and hypnotic activity in rats described for this compound (Di Fabio et al., 2011). GSK-1059865 is a selective OX1R antagonist that shows a 80 fold higher selectivity vs. OX2R (Gozzi et al., 2011). We confirmed the high selectivity of GSK-1059865 (pKi OX1R = 8.3, pKi OX2R = 6.4; unpublished data). GSK-1059865 (10 mg/kg) achieved about 90% OX1R occupancy 15 min after subcutaneous (sc) administration which was maintained for 4 h, as determined by *ex vivo* autoradiography in the rat brain (manuscript in preparation). JNJ-10397049 is a selective OX2R antagonist (pKi OX2R = 8.2, pKi OX1R = 5.7). JNJ-10397049 (30 mg/kg) achieved about 80% of OX2R occupancy 15 min after sc administration which was maintained for 6 h, as determined by *ex vivo* autoradiography in rat cortex (Dugovic et al., 2009). SB-649868, JNJ-10397049, and GSK-1059865 were synthesized at Janssen Research & Development, L.L.C. SB-649868 (10 and 30 mg/kg) was dosed orally as a suspension of 0.5% methylcellulose in a volume of 1 ml/kg. JNJ-10397049 (10 mg/kg) and GSK-1059865 (10 mg/kg) were administered via the subcutaneous (sc) route. GSK-1059865 and JNJ-10397049 were formulated in 5% pharmasolve, 20% solutol, 75% hydroxypropyl-β-cyclodextrin (20% w/v), and were injected as a free base form of the compound in a volume of 1 ml/kg.

The dose-response experiment with SB-649868 was carried out in a group of animals (*n* = 8) assigned to three treatment conditions (vehicle, *n* = 8; 10 mg/kg, *n* = 7; 30 mg/kg, *n* = 7). The experiment with the simultaneous coadministration of GSK-1059865 and JNJ-10397049 was carried out on a separate group of rats (*n* = 7) assigned to four treatment conditions (vehicle + vehicle, GSK-1059865 + vehicle, vehicle + JNJ-10397049, and GSK-1059865 + JNJ-10397049). Both experiments were conducted in a randomized cross-over design and a minimum of 3 days washout period were allowed between two treatments.

### Sleep recording and analysis

Animals were implanted with telemetric devices for polysomnographic recording of sleep-wake patterns as previously described (Dugovic et al., 2009). To determine states of vigilance, polysomnographic waveforms were acquired from two stainless steel screw electrodes that were implanted under isofluorane anesthesia in the frontal and parietal cortex for the electroencephalogram (EEG) and in dorsal nuchal muscles for the electromyogram (EMG). Electrodes were coupled to a sterile two-channel telemetric device (PhysioTel F40-EET; Data Sciences International, St. Paul, MN) that had been implanted in the intraperitoneal cavity in order to acquire measurements of body temperature and locomotor activity. After a 2-week period of recovery from surgery, animals were transferred to their designated housing/procedure room to allow for adaptation to the recording chamber and environment.

EEG and EMG signals were recorded for up to 12 h post-drug administration and were digitized at a sampling rate of 100 Hz on an IBM PC-compatible computer using Dataquest A.R.T software (Data Sciences International). Using the computer software program SleepSign (Kissei Comtec, Nagano, Japan), consecutive EEG/EMG recordings were divided into individual 10 sepochs that were then visually assigned vigilance states based upon conventional criteria for wake, NREM sleep and REM sleep as described previously. EEG activity within specific vigilance states was determined by power spectral analysis using the Fast Fourier Transform performed within a frequency range of 1–30 Hz. Values for power spectra were divided into four frequency bands: delta (1–4 Hz), theta (4–10 Hz), sigma (10–15 Hz), and beta (15–30 Hz).

Analysis of sleep-wake parameters included latency (onset) to NREM sleep (defined as the time interval to the first six consecutive NREM epochs) and REM sleep (the first two consecutive REM epochs post-injection), the duration of wake, NREM and REM sleep and bout analysis (number and duration) for each vigilance state. In addition, episodes of direct wake to REM sleep (DREM) transitions were assessed. A DREM transition was defined as an abrupt episode of nuchal atonia and EEG dominance of theta activity lasting at least a 10 s epoch with at least six consecutive 10 s epochs of wake (60 s) preceding the episode. As a comparison, a criteria of at least 40 s of wakefulness preceding an episode of cataplexy has been defined in mouse models of narcolepsy (Scammell et al., 2009).

Results were averaged and expressed as mean ±s.e.m. in defined time intervals. To determine whether differences were significant at a given interval, either a One-Way analysis of variance (ANOVA) with Newman–Keuls or Dunnett's multiple comparison *post-hoc* analysis, or Two-Way repeated measures ANOVA followed by a Bonferroni *post-hoc* test was performed.

## Results

### Differential NREM and REM sleep-promoting effects of the dual OX1/2R antagonist SB-649868

**Figure 1:**
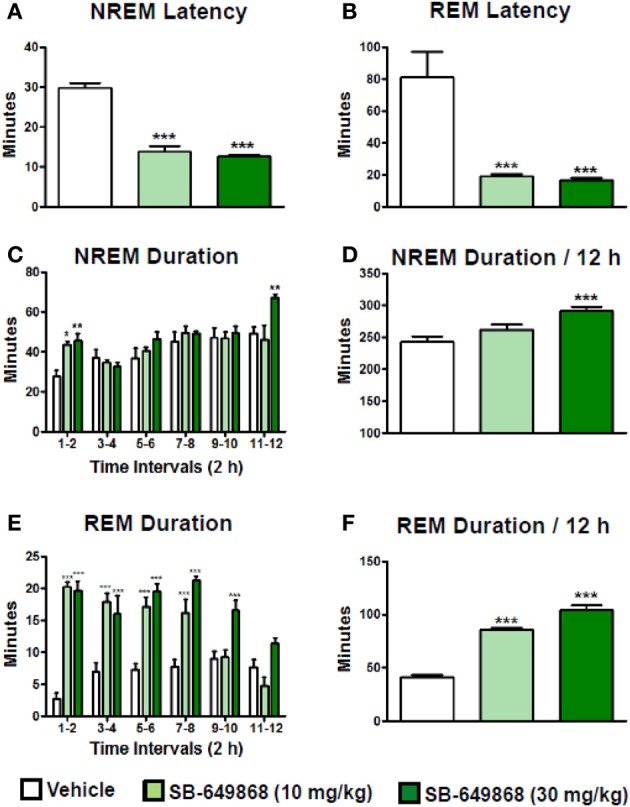
**Sleep-promoting effects of the dual OX1/2R antagonist SB-649868 in rats.** Latency to NREM **(A)** and REM **(B)** sleep and duration of NREM **(C,D)** and REM **(E,F)** sleep during the 12-h dark phase after oral dosing (10 and 30 mg/kg) are expressed in minutes and are represented as means ± s.e.m. (*n* = 7–8 animals per condition). ^*^P < 0.05, ^**^P < 0.01, and ^***^P < 0.001 vs. vehicle, based on One-Way ANOVA followed by Dunnett's multiple comparison *post-hoc* test **(A)** [*F*_(2, 19)_ = 70.60, *p* < 0.001], **(B)** [*F*_(2, 19)_ = 14.05, *p* < 0.001], **(D)** [*F*_(2, 19)_ = 9.57, *p* = 0.001], and **(F)** [*F*_(2, 19)_ = 127.50, *p* < 0.001] or two-way ANOVA (interaction Time × Treatment) followed by Bonferroni *post-hoc* test **(C)** [*F*_(10, 144)_ = 2.45, *p* = 0.011] and **(E)** [*F*_(10, 114)_ = 8.07, *p* < 0.001].

**Figure 2:**
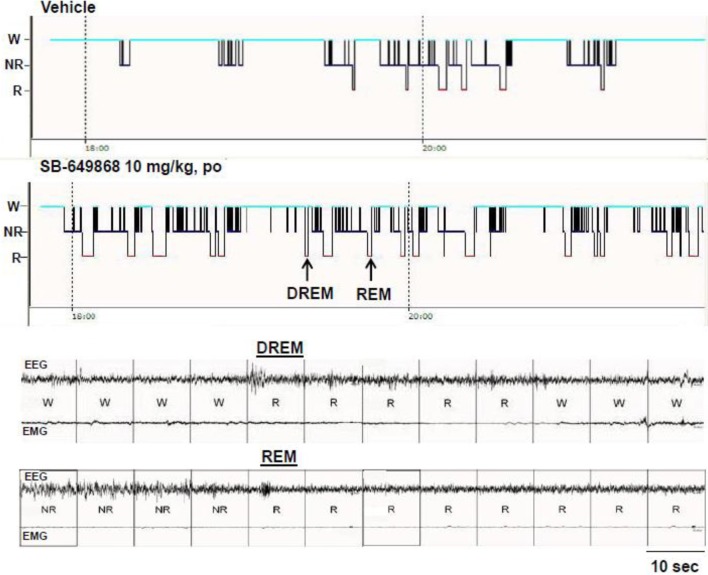
**Representation of DREM in an animal after administration of the OX1/2R antagonist SB-649868.** The arrows on the hypnogram represent the respective DREM and REM episodes illustrated on corresponding EEG/EMG traces.

When administered at the onset of the dark phase, the dual OX1/2R antagonist SB-649868 significantly reduced the latencies for NREM and REM sleep, and significantly prolonged the time spent in each of these sleep states at the two doses tested, 10 and 30 mg/kg (Figure 1). However, REM sleep was predominantly affected relative to NREM sleep in regard to its onset and total duration. The latency for NREM sleep was reduced by half (Figure 1A), whereas the latency for REM sleep was diminished by about 75% (Figure 1B) as compared to the vehicle condition, leading to an almost similar onset for both states. Similarly, the time course of the effects on REM sleep differed from the effects on NREM sleep. The increase in NREM sleep duration occurred mostly during the first 2 h after the treatment (Figure 1C), whereas the increase in REM duration lasted for 8 h at the 10 mg/kg dose and for the entire 12-h dark phase following the dose of 30 mg/kg (Figure 1E). In the total 12-h period, the increase in NREM sleep duration was significant at the high dose only (Figure 1D) whereas REM sleep was significantly increased at both doses tested (Figure 1F). Further analysis of the sleep macrostructure showed that while the numbers of both NREM and REM bouts were dose-dependently enhanced, the NREM bout duration was reduced but the REM bout duration was prolonged (Table 1). Therefore, the net increase in the time spent in NREM sleep might be attenuated due to a decrease in NREM sleep continuity in spite of the increase in its frequency. In contrast, both the frequency and continuity in REM sleep were increased, leading to a larger increment in the total REM sleep duration. In addition, a careful visual analysis of the EEG and EMG signals revealed scarce episodes of direct transitions from wake to REM sleep (DREM). DREM episodes occurred in 3 out of 7 rats treated with the highest dose of 30 mg/kg, and in one animal which received the dose of 10 mg/kg as illustrated in the hypnogram and the EEG/EMG traces corresponding to this event (Figure 2). Power spectral analysis indicates that the averaged EEG relative power in the theta frequency band (4–10 Hz) during this DREM episode (45% of the total power) was comparable to the relative theta power during a normal episode of REM sleep (49%) shown in Figure 2 for this animal. The averaged EEG theta activity contributed to 26% of the total power during the 40 s wake episode preceding DREM and to 31% during the 50 s wake episode following DREM, indicating a distinct range of power density values compared to the theta activity during DREM.

**Table 1:** **NREM and REM bout analysis after oral administration of the dual OX1/2R antagonist SB-649868 in rats**.

	**NREM bout number**	**NREM bout duration (min)**	**REM bout number**	**REM bout duration (min)**
Vehicle	154.4±4.8	1.57±0.04	31.9±2.5	1.32±0.07
10 mg/kg	211.1±9.1^***^	1.22±0.07^***^	46.7±2.8^**^	1.85±0.09^**^
30 mg/kg	248.4±7.6^***^	1.15±0.04^***^	52.0±3.2^***^	2.02±0.13^***^

Values (means ± s.e.m. n = 7–8 animals per condition) are calculated for the 12-h dark phase after compound administration.

** P < 0.01 and

*** P < 0.001 vs. Vehicle based on one-way ANOVA followed by Dunnett's multiple comparison post-hoc test.

*NREM Bout Number [F*_(*2, 19*)_ = *44.58, p* < *0.001]; NREM Bout Duration [F*_(*2, 19*)_ = *19.38, p* < *0.001]; REM Bout Number [F*_(*2, 19*)_ = *14.10, p* < *0.001]; REM Bout Duration [F*_(*2, 19*)_ = *13.94, p* < *0.001].*

### Disinhibition of REM sleep by additional pharmacological blockade of OX1R to OX2R antagonism

**Figure 3:**
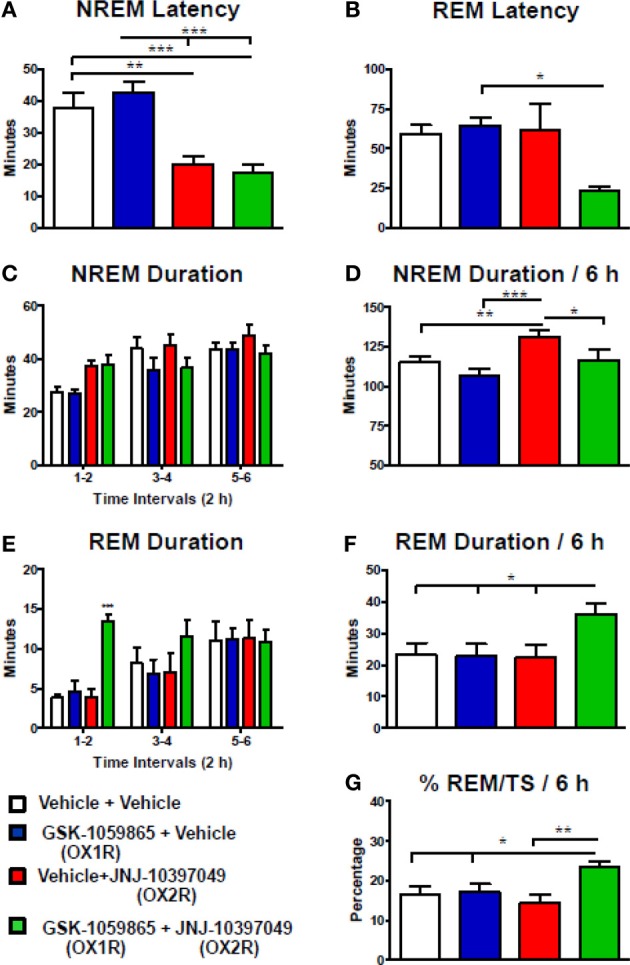
**Desinhibition of REM sleep in rats by additional pharmacological blockade of OX1R to OX2R antagonism.** NREM **(A)** and REM **(B)** sleep latency, duration of NREM **(C,D)** and REM **(E,F)** sleep, and % REM/TS **(G)** were determined for 6 h after the coadministration of GSK-1059865 (10 mg/kg) and JNJ-10397049 (10 mg/kg) at dark onset. Values (means ± s.e.m. *n* = 7 animals) are expressed in minutes (except **G**). Statistical significance (^*^P < 0.05, ^**^P < 0.01, and ^***^P < 0.001) was based on repeated measures one-way ANOVA followed by Newman–Keuls *post-hoc* test **(A)** [*F*_(3, 6)_ = 17.87, *p* < 0.001], **(B)** [*F*_(3, 6)_ = 3.72, *p* = 0.03], **(D)** [*F*_(3, 6)_ = 11.59, *p* < 0.001], **(F)** [*F*_(3, 6)_ = 5.59, *p* = 0.006], and **(G)** [*F*_(3, 6)_ = 7.41, *p* = 0.002] or repeated measures two-way ANOVA (interaction Time × Treatment) followed by Bonferroni *post-hoc* test **(C)** [*F*_(6, 48)_, = 1.27, *p* = 0.288] and **(E)** [*F*_(6, 48)_ = 2.78, *p* = 0.021].

To further investigate the differential actions of the dual OX1/2R antagonist on NREM and REM sleep, rats were administered either with the selective OX1R antagonist GSK-1059865 (10 mg /kg) or the selective OX2R antagonist JNJ-10397049 (10 mg/kg) alone, or in combination at the onset of the dark phase. The results were presented in Figure 3 for the first 6-h period after dosing, based on the shorter duration of both the sleep-promoting effect of JNJ-10397049 and the sleep response elicited by simultaneous injection of GSK-1059865 at the respective doses tested. Sleep-wake parameters were not affected during the second 6-h period (data not shown). Administration of the OX2R antagonist alone induced a significant reduction in NREM sleep latency (Figure 3A) and an increase in NREM sleep duration (Figures 3C,D) relative to vehicle treatment. While the OX1R antagonist had no effect on NREM sleep by itself, its administration significantly attenuated the NREM sleep prolongation evoked by the OX2R antagonist (Figures 3C,D). Indeed, in the combined treatment condition the NREM bout duration was significantly reduced relative to all other conditions (Table 2), accounting for the less pronounced increment in total NREM sleep duration. The time course analysis shows that the reduced effect on NREM sleep occurred 2 h after the additional administration of GSK-1059865 (Figure 3C), and consequently the NREM sleep latency was not affected (Figure 3A). REM sleep onset (Figure 3B) and REM sleep duration (Figures 3E,F) were not altered by either OX1R or OX2R pharmacological blockade. In contrast, when receiving the combined treatment the animals displayed a reduced REM sleep latency (Figure 3B) and the time spent in REM sleep was significantly increased as compared to treatment with vehicle, OX1R or OX2R antagonist alone (Figures 3E,F). This REM sleep-promoting effect was observed mainly during the first 4 h following the treatment (Figure 3E) and was due to a significant prolongation of the REM bout duration as well as a tendency to enhanced REM bout numbers (Table 2). Ultimately, the results showed that additional pharmacological blockade of OX1R attenuated the NREM sleep-promoting effects of an OX2R antagonist by increasing REM sleep duration and concomitantly decreasing NREM sleep duration, leading to a significant enhancement of the REM/total sleep ratio (% REM/TS) as illustrated in Figure 3G. Similarly, a markedly elevated % REM/TS was found following the administration of the dual OX1/2R antagonist SB-649868 at the doses of 10 mg/kg (24.6%) and 30 mg/kg (26.4%) as compared to vehicle treatment (14.6%) over the 12-h dark phase. Therefore, both experimental approaches produced a disinhibition of REM sleep by shifting the balance in favor of REM sleep at the expense of NREM sleep. However, unlike with SB-649868, no DREM episodes were detected with the coadministration of GSK-1059865 and JNJ-10397049.

**Table 2:** **NREM and REM bout analysis after co-administration of the OX1R antagonist GSK-1059865 (10 mg/kg) and the OX2R antagonist JNJ-10397049 (10 mg/kg) in rats**.

	**NREM bout number**	**NREM bout duration (min)**	**REM bout number**	**REM bout duration (min)**
Vehicle + Vehicle	82.7±2.6	1.39±0.07	19.6±2.0	1.13±0.13
GSK-1059865 + Vehicle	70.7±3.7^*^^a^	1.51±0.10^c^	18.1±1.7	1.21±0.17
Vehicle + JNJ-10397049	101.0±4.2^**^	1.30±0.07	17.7±3.6	1.37±0.18
GSK-1059865 + JNJ-10397049	97.9±3.0^*^	1.17±0.07^*^^,^^b^	23.0±2.1	1.55±0.11^**^^,^^b^

Values (means ± s.e.m. n = 7 animals) are calculated for the 6-h period following the treatment.

* P < 0.05 and

** P < 0.01 vs. Vehicle + Vehicle

a P < 0.001 vs. Vehicle + JNJ-10397049 and GSK-1059865 + JNJ-10397049

b P < 0.01 vs. GSK-1059865 + Vehicle

c P < 0.05 vs. Vehicle + JNJ-10397049

As determined by repeated measures One-Way ANOVA followed by Newman–Keuls post-hoc test.

*NREM Bout Number [F*_(*3, 6*)_ = 13.69, *p* < *0.001]; NREM Bout Duration [F*_(*3, 6*)_ = *7.79, p* = *0.001];*

*REM Bout Number [F*_(*3, 6*)_ = *1.28, p* = *0.313]; REM Bout Duration [F*_(*3, 6*)_ = *6.76, p = 0.003].*

## Discussion

The present investigation demonstrated that pharmacological blockade of both OX1R and OX2R is effective in promoting both NREM and REM sleep but produced an alteration of the sleep stages distribution due to a striking impact on REM sleep. The dual OX1/2R antagonist SB-649868 primarily reduced REM sleep latency and prolonged REM sleep time in comparison to a less pronounced NREM sleep-promoting effect. Similarly, administration of a selective OX1R antagonist in combination with a selective OX2R antagonist exclusively enhanced REM sleep by counteracting the NREM sleep-promoting effects evoked by the OX2R blockade. Therefore, transient pharmacological inhibition of the two receptors, either by a dual OX1/2R antagonist or by simultaneous blockade of OX1R to OX2R antagonism, disrupted sleep architecture by shifting the balance in favor of REM sleep at the expense of NREM sleep.

Selective pharmacological blockade of OX2R by JNJ-10397049 promotes sleep by inhibiting the output of wake active neurons, mainly by suppressing histamine release in the hypothalamus (Dugovic et al., 2009). Conversely, orexin-A administration increased cortical histamine release without affecting the norepinephrine or serotonin release in mice (Hong et al., 2005). The robust hypnotic properties of several selective OX2R antagonists after systemic administration in rats and mice have been confirmed by other groups (Gozzi et al., 2011; Morairty et al., 2012; Betschart et al., 2013). In these dose-response studies, results showed that NREM sleep was firstly increased at low dosage and that REM sleep was progressively enhanced at higher doses, with no obvious change in the REM/TS ratio indicating a preservation of the sleep architecture. By contrast to selective OX2R antagonists, pharmacological (using various OX1R antagonists with distinct chemical structures) or genetic selective inhibition of OX1R in rodent models has been reported to minimally affect sleep-wake states in baseline conditions (Smith et al., 2003; Sakurai, 2007; Dugovic et al., 2009; Gozzi et al., 2011). Controversial data has been reported in one study with the OX1R antagonist SB-334867 (Morairty et al., 2012) which is less selective and less potent than GSK-1059865 and exhibits off target activities (Gotter et al., 2012). Our results confirm the absence of sleep-promoting effects of GSK-1059865 previously reported by Gozzi et al. (2011), and a more recent study showed that the new selective OX1R antagonist ACT-335827 did not alter sleep in rats (Steiner et al., 2013). However, due to the paucity of publically available selective orexin compounds, SB-334867 remains so far the most studied OX1R antagonist and has been found to reverse the arousal and REM sleep suppression induced by pharmacological (orexin-A injection) or optogenetic activation of orexin neurons through OX1R in the locus coeruleus (Bourgin et al., 2000; Smith et al., 2003; Carter et al., 2012).

Within the last decade, dual OX1/2R antagonists have been developed as therapeutics for insomnia and their hypnotic properties have been demonstrated in animals, human volunteers, and insomnia patients. The development of the former compounds almorexant and SB-649868 has been stopped for undisclosed adverse effects; suvorexant is in the latest stage of clinical development and is followed by its back up compound filorexant (Winrow and Renger, 2014). In the present study, rats treated with SB-649868 at the onset of their active/dark phase displayed a markedly reduced REM sleep latency and the first episode of REM sleep was observed shortly after NREM sleep onset (Figure 1). While both sleep stages were enhanced, the predominant increase in REM sleep was reflected by the abnormally elevated REM/TS ratio compared to vehicle treatment. These data are in agreement with those previously found in rats when SB-649868 was dosed in the middle of the dark phase (Di Fabio et al., 2011). A similar increase in the proportion of REM vs. NREM sleep has been reported in mice dosed with almorexant or suvorexant (Mang et al., 2012; Betschart et al., 2013; Black et al., 2013), although not in rats (Brisbare-Roch et al., 2007; Winrow et al., 2011).

Our visual examination of EEG/EMG recordings revealed the occurrence of at least one episode of REM intrusion into wake (DREM) in 4 out of 7 animals dosed with SB-649868 (Figure 2). Simultaneous video recordings were not performed, therefore the behaviors associated with this activity during this unusual state transition in these rats are unclear. However, we did not observe any DREM event with the coadministration of the OX1R antagonist GSK-1059865 and the OX2R antagonist JNJ-10397049. While both compounds exhibit efficient brain-penetrating properties (see *ex vivo* receptor occupancy in methods), the REM sleep promotion produced by SB-649868 was much more pronounced and long-lasting as compared to the combined treatment, that might increase the possibility to trigger DREM transitions. In a murine model of narcolepsy, the orexin/ataxin-3 transgenic mouse, almorexant exacerbated spontaneous cataplexy, and possibly elicited cataplexy-like events in some wild type mice after wheel running activity (Black et al., 2013). In a preliminary investigation conducted in mice deficient for the OX2R, we also observed episodes of DREM following the treatment with another dual OX1/2R antagonist (Dugovic et al., 2012). Mice lacking both orexin receptors or the orexin peptide exhibit some cataplexy spontaneously (Sakurai, 2007) that can be substantially increased by pleasurable activity such as wheel running or eating highly palatable foods (Espana et al., 2007; Clark et al., 2009; Oishi et al., 2013). Cataplexy, a pathological intrusion of REM sleep atonia into wakefulness, has not been reported with almorexant or suvorexant in clinical or preclinical studies in situations where cataplexy is not provoked (Brisbare-Roch et al., 2007; Winrow and Renger, 2014). However, there are no disclosed clinical trials with an OX1/2R antagonist under conditions of positive emotional stimuli that are known to trigger cataplexy in narcolepsy with cataplexy patients.

Narcoleptic patients also exhibit sleep onset REM (SOREM) episodes usually defined as REM sleep latency shorter than 15 min (Nishino and Mignot, 1997). After SB-649868 administration, SOREM episodes were observed in Phase I studies (Bettica et al., 2012a), in a model of situational insomnia in healthy volunteers (Bettica et al., 2012b), as well as in patients with primary insomnia (Bettica et al., 2012c). Although SOREM episodes were not detected after administration of almorexant or suvorexant in primary insomnia patients, it is noteworthy that the latency for REM sleep was significantly reduced at half the dose required to shorten the latency to persistent sleep for both compounds (Herring et al., 2012; Hoever et al., 2012). In patients with primary insomnia treated with suvorexant or SB-649868, the increase in total sleep time resulted from a higher percentage of time spent in REM sleep and to a lesser degree in stage-2 sleep (Bettica et al., 2012c; Herring et al., 2012). Therefore, the preferential sleep-promoting action of dual OX1/2R antagonists on REM sleep relative to NREM sleep in animal studies seems to be predictive of the alterations in sleep architecture observed in humans.

The results of the experiment conducted in rats receiving the selective OX1R antagonist GSK-1059865 and the selective OX2R antagonist JNJ-10397049 in combination were consistent with the data obtained with the dual OX1/2R antagonist SB-649868. While REM sleep was not affected either by the OX2R antagonist or the OX1R antagonist alone, their coadministration reduced REM sleep latency and prolonged REM sleep time. Concurrently, the magnitude of the NREM sleep promoting effect elicited by the OX2R blockade was attenuated, demonstrating a shift in the balance between NREM and REM sleep. In a previous investigation carried out during the light/rest phase of the rat using the same selective OX2R antagonist, but with the OX1R antagonist SB-408124 which displayed less brain penetration (Gotter et al., 2012), we mainly observed a diminution in NREM sleep with the combined treatment vs. the OX2R antagonism alone (Dugovic et al., 2009). Together, these data indicate that additional OX1R blockade attenuated the NREM sleep promoting effect of an OX2R antagonist by disinhibiting REM sleep likely through OX1R.

In summary, we demonstrated that OX1R blockade dysregulates REM sleep in the presence of OX2R antagonism. These findings reinforce the consensus based on various animal models that wake to NREM sleep transitions depend on OX2R signaling and that REM sleep dysregulation occurs by the loss of both OX1R and OX2R function (Willie et al., 2003; Mieda et al., 2011; Mochizuki et al., 2011), thereby confirming the distinct contribution of OX1R and OX2R in the control of sleep-wake states. Key insights recently gained from the above clinical studies suggest that transient blockade of orexin receptors by dual OX1/2R antagonists induce a preferential disinhibition of REM sleep relative to NREM sleep, and may cause a dysregulation of REM sleep. Since the blockade of OX2R is sufficient to initiate and promote sleep in animals, future clinical studies with selective OX2R antagonists should answer the question of whether this hypothesis is translatable to humans.

## Author contributions

Christine Dugovic designed research, analyzed data, and wrote manuscript; Jonathan E. Shelton analyzed data and edited manuscript; Sujin Yun conducted research and analyzed data; Pascal Bonaventure participated in research design and edited manuscript; Brock T. Shireman provided compounds; Timothy W. Lovenberg participated in research design.

### Conflict of interest statement

All the authors are full-time employees of Janssen Research & Development, L.L.C.
